# O-32 - WHO BENEFITS MOST? INFLUENZA AND COVID VACCINATION, HOSPITALISATION RISK, AND SOCIOECONOMIC DEPRIVATION

**DOI:** 10.1093/pubmed/fdag046.028

**Published:** 2026-07-09

**Authors:** Mr Safraj Shahul Hameed, Dr Kirsty Morrison, Prof Chris Robertson, Dr Ross McQueenie, Dr Kimberley Marsh

**Affiliations:** Public Health Scotland; Public Health Scotland; Public Health Scotland; University of Strathclyde; Public Health Scotland; Public Health Scotland

## Abstract

**Background:**

Seasonal influenza and COVID19 remain major contributors to winter hospitalisations. Vaccination can reduce severe respiratory disease, yet uptake is lowest in the most deprived communities, raising equity concerns. Understanding how vaccination and deprivation affect the risk of acute respiratory hospitalisations is essential to developing strategies to reduce health inequalities.

**Objectives:**

To estimate acute respiratory infection (ARI) hospitalisation rates by influenza and COVID19 vaccination status and socioeconomic deprivation and to quantify hospitalisations averted under different uptake scenarios.

**Methods:**

A retrospective cohort study was conducted among Scottish adults aged 65 years and older during the 2024/25 season. Emergency ARI hospitalisation was identified using ICD10 codes. Vaccination status was classified as unvaccinated, influenza only, COVID19 only, or both, with exposure defined from 14 days postvaccination. Deprivation was measured using Scottish Index of Multiple Deprivation quintiles. Cox proportional hazards models adjusted for age, sex, and clinical risk were fitted, including interaction terms. Scenario modelling estimated hospitalisations averted under actual, no and universal vaccination uptake.

**Results:**

ARI hospitalisation rates showed a strong socioeconomic gradient, highest in the most deprived (32.2 per 1,000 person-years) and lowest in the least deprived groups (14.4). Vaccination reduced hospitalisation risk across all deprivation levels, with combined influenza and COVID-19 vaccination offering the greatest protection, and no evidence that deprivation level altered vaccine effectiveness. Scenario modelling showed more hospitalisations averted in absolute terms in the most deprived groups (956 vs 784), while relative reductions due to vaccination were greater in the least deprived (27.8% vs 22.4%). Even with universal uptake, absolute inequalities in hospitalisation persisted.

**Conclusions:**

Vaccination protects against ARI hospitalisation across socioeconomic groups, but unequal uptake and baseline risk result in unequal population impact. Improving vaccine uptake in deprived populations can reduce, but not eliminate, inequalities. Vaccination alone is insufficient to address persistent inequalities; wider action to address social and structural determinants is required.

